# Latest Trichin-Epidemics in Germany*Zeitschrift für Mikroskopie und Fleischschau. Jan. 5 and 20, 1884.

**Published:** 1884-04

**Authors:** 


### THE LATEST TRICHIN-EPIDEMICS IN GERMANY.*

### (Emersleben, Deesdorf, Nienhagen and Thorn.)

THESE villages are all situated in Saxony. Emersleben has about 760 inhabitants. It has been long the custom of many of the people here to eat *raw hashed pork* with their bread. The late outbreak of Trichiniasis was due to the same cause. On the 14th and 15th of September, 1883, a large amount of this *raw hashed pork* was sold and consumed; it was all purchased of one butcher. The first appearance of disease occurred at about the same time (the second day after eating the pork) among several individuals, the symptoms being nausea emesis and diarrhœa. The first case that appeared for treatment was on September 20th, and the last on the 15th of October. The latter was an old woman (63) that would not at first listen to any proposals for medical attendance, but finally consented after a son and daughter had died from the disease.

The first symptoms of the disease, aside from those already mentioned, were pain in the stomach and intestines, great thirst, fever, temperature 38° to 41° C. The inclination to emesis disappeared within a few days, although the diarrhœa continued, but in a less severe degree up to the fifth or seventh day; at this period the patients suffered intensely with stiffness

*Zeitschrift für Mikroskopie und Fleischschau. Jan. 5 and 20, 1884.

and pains in the limbs, especially severe on movement or pressure, one of the chief phenomena being *œdema of the eyelids and face,* which becomes of great diagnostic value when occurring in connection with the previously mentioned symptoms. The condition remained unchanged until *typhoid* phenomena began to be demonstrated, such as sleeplessness, the tongue being parched, cracked and covered with a brown coating, further delirium, and instead of diarrhœa, constipation. Many cases were complicated with pneumonia, pleurisy, meningitis, herpes and aphthous eruptions. The œdema was sometimes very severe, leading to erosions and bed sores. Where the tongue, pharynx and larynx were severely invaded, the patients suffered intense agonies both from the difficulty of swallowing and to obtain breath from the painful and swollen condition of the tissues. The first death occurred on the 3d of October in a person that had eaten the meat on the 15th of September. The autopsy revealed the presence of an immeasurable quantity of intestinal trichinae, as well as an intense invasion of the diaphragmatic and other muscles. The second death occurred on the 5th of October, and they then followed in rapid succession; the greatest number occurring in the fifth and sixth week of the disease, there being eleven reported for the former and ten for the latter. The number of cases reported was 257, of whom 50 have already died. The oldest person that died was 76, the youngest 12 years old. Many children were diseased, the youngest being one and a quarter years old, but with the exception of the 12 year old boy, fortunately recovered. Of the 50 that died, 20 were males and 30 females. There were many cases in which the disease did not have as severe a form, from the persons having eating the meat in a cooked (?) condition. They suffered some diarrhœa and pains in the limbs, and some œdema, but were not confined to their bed.

The butcher that killed the hog and sold it, as well as his wife, were both very sick, as well as the person that was supposed to have examined the meat for trichinæ.

No apparently beneficial results seemed to follow the most varied medical treatment.

The entire members of three families died from the disease; while of another family four persons died, and of another three.

Deesdorf contains 650 inhabitants. Of these forty or more became diseased, and nine or ten died.

Nienhagen has 350 inhabitants, of whom eighty had trichinosis at the same time, but only one died.

The inhabitants of these three villages are all principally supplied with meat by *one butcher*, and the same examiner is appointed for all of them.

The first case occurred at Thorn on the 11th of November, 1883, among some 60 persons, and although many of them continued to suffer for a long time, in no case did the disease terminate fatally.

This, as well as every other invasion, was due to carelessness in inspection in the first place, and in cooking in the second. B.

